# Discovering the Potential of High Phonon Energy Hosts in the Field of Visible-to-Ultraviolet C Upconversion

**DOI:** 10.1021/acs.jpclett.4c02053

**Published:** 2024-09-06

**Authors:** Patrycja Zdeb, Nadiia Rebrova, Przemysław J. Dereń

**Affiliations:** Institute of Low Temperature and Structure Research, Polish Academy of Science, ul. Okólna 2, 50-422 Wrocław, Poland

## Abstract

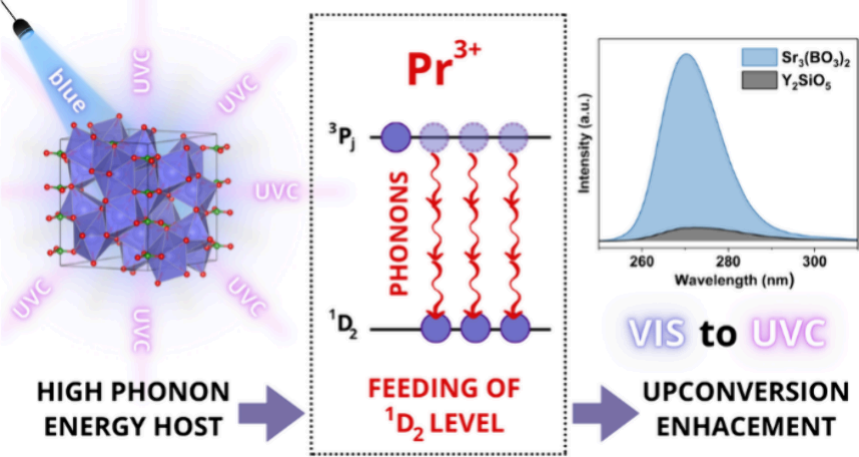

The recent pandemic has intensified the search for new ultraviolet C (UVC) phosphors excited by low-intensity visible light that can be used for disinfection or therapeutic purposes. Currently, the most promising phosphor with efficient upconversion was thus far Y_2_SiO_5_ (YSO) doped with Pr^3+^. However, we have studied a new material Sr_3_(BO_3_)_2_:Pr^3+^ (SBO), whose upconversion emission is 10 times stronger than YSO, despite the high phonon energy possessed by SBO and despite the lack of optimization of synthesis and dopant concentration and the absence of co-dopant that should be added to compensate for the charge. Such an efficient upconversion is achieved by engagement of the ^1^D_2_ level, which is populated by both the multiphonon non-radiative transition and the closed feedback loop. From this level, blue excitation can reach the 4f^1^5d^1^ electronic configuration. At the same time, due to a small Stokes shift, the 5d levels emit exclusively in the UVC and partially in the ultraviolet B (UVB) region.

Obtaining a material that would efficiently convert visible radiation into ultraviolet C (UVC) is a dream of many groups. Such a material could be excited by solar radiation, paving the way for the construction of many new devices that operate without additional electrical energy, e.g., drinking water treatment systems and systems for producing green hydrogen from water. It could also be used in therapy to destroy cancer or dysplastic cells. Thus far, such a promising and most efficient material that converts low-energy exciting photons into UVC by upconversion seems to be Y_2_SiO_5_ (YSO) doped with Pr^3+^ ions.^1−3^ Cates and colleagues showed that upconversion is observed even at low excitation radiation intensities.^1^

In our letter, we report the exciting discovery of a new material whose upconversion emission intensity is 10 times stronger than in YSO. Research efforts have thus far focused on finding and investigating materials with low phonon energy to ensure the long enough occupation of the intermediate level because the efficiency of upconversion is proportional to the fifth power of the lifetime of this level. Therefore, many works on obtaining such emissions concerned fluorides.^4−6^ The use of borates as a search matrix seems to be in total contradiction with this idea. Their phonon energy of about 1400 cm^–1^ ^7^ leads to an efficient level bridging multiphonon non-radiative transitions and effectively reducing the intermediate-level lifetime of the dopant emission. However, in Sr_3_(BO_3_)_2_ (SBO), it turned out to be an advantage; in the following paragraphs, we will describe the role of phonons in the visible-to-UVC upconversion process and emphasize the most effective approach for developing efficient UVC upconverters.

*Materials and Synthesis*. Sr_3_(BO_3_)_2_:*x* mol % Pr^3+^ (*x* = 0.1, 0.25, 0.5, 0.75, 1, 1.5, 2, 3, 5, and 7) phosphor was prepared by high-temperature solid-state reaction. The stoichiometric amounts of SrCO_3_ (99.9%) and Pr_2_O_3_ (99.9%) along with a 5% excess of H_3_BO_3_ were mixed carefully in an agate mortar for approximately 10 min. The resulting mixture was then transferred to corundum crucibles and pre-annealed at 500 °C for 3 h. The samples were mixed again and subjected to annealing at 1000 °C for 10 h in an air atmosphere. After the samples were annealed, the powders were ground and collected for further analysis. Excess boric acid was used to prevent losses due to evaporation during the annealing process.

*Sample Characterization*. Emission spectra and decay profiles of Stokes processes were performed with a FLS1000 fluorescence spectrometer (Edinburgh Instruments), equipped with a 450 W ozone-free xenon lamp and a xenon flash lamp. The measured spectra were corrected for the sensitivity of the spectrophotometers. The upconversion luminescence of the samples was recorded using a McPherson model 218 high-resolution scanning monochromator (300 mm) with continuous diode laser excitation at a wavelength of 444 nm. All samples were measured under identical conditions using a UG5 optical filter and a solar-blind photomultiplier (Hamamatsu R7154P). The diode laser beam was focused with a lens of 20 cm focal length onto a rectangular spot (1 × 1.5 mm).

Pr^3+^-doped SBO shows a very strong UVC Stokes emission. Excited at about 246.5 nm, it exhibits almost only 4f^1^5d^1^ → 4f^2^ emission, with the maximum at 270 nm (see Figure 1). Extremally weak emission corresponding to the 4f^2^ → 4f^2^ transitions occurs in the visible range and overlaps with the broad 4f^1^5d^1^ → 4f^2^ bands. Due to the small Stokes shift, which we calculated to be only 2230 cm^–1^, the 5d minimum of the configuration parabola is not displaced much from the equilibrium position of the 4f configuration parabola,^8^ and the crossover quenching of 4f5d emission is not effective. This is very important for the utility reasons of this phosphor, because the emission channel in the visible range would be an unnecessary waste of the excitation energy.

**Figure 1:**
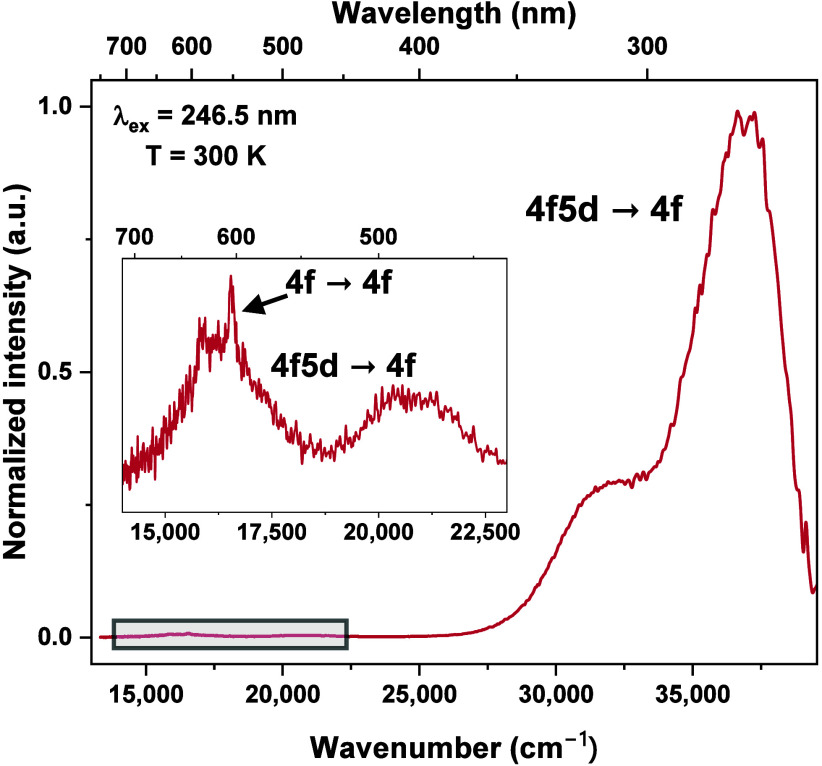
Room-temperature (RT) emission spectrum of SBO:1% Pr^3+^ excited at 246.5 nm. The inset presents the part of the spectrum marked with the gray frame. Narrow bands in the inset correspond with the 4f → 4f transitions, and the broad bands are associated with the 4f5d → 4f transitions.

A 444 nm laser excitation generates an UVC upconversion emission, the same as the Stokes excitation at 246.5 nm (Figure 2a). Comparing the upconversion luminescence intensities of SBO and YSO, we found the 10-fold enhancement in the upconversion (UC) for borate lattice for a 4 times smaller Pr^3+^ molar concentration. Moreover, 84% of the radiation emitted by SBO falls into the UVC range; for YSO, it is only 64%, making SBO a better germicidal agent. The dependence of the UVC UC emission intensity upon the power shows an interesting feature (Figure 2b); initially, from low excitation power density values up to 35 W/cm^2^, the slope of the line passing through the experimental points is 2, indicating the involvement of two photons to obtain the UC.

**Figure 2:**
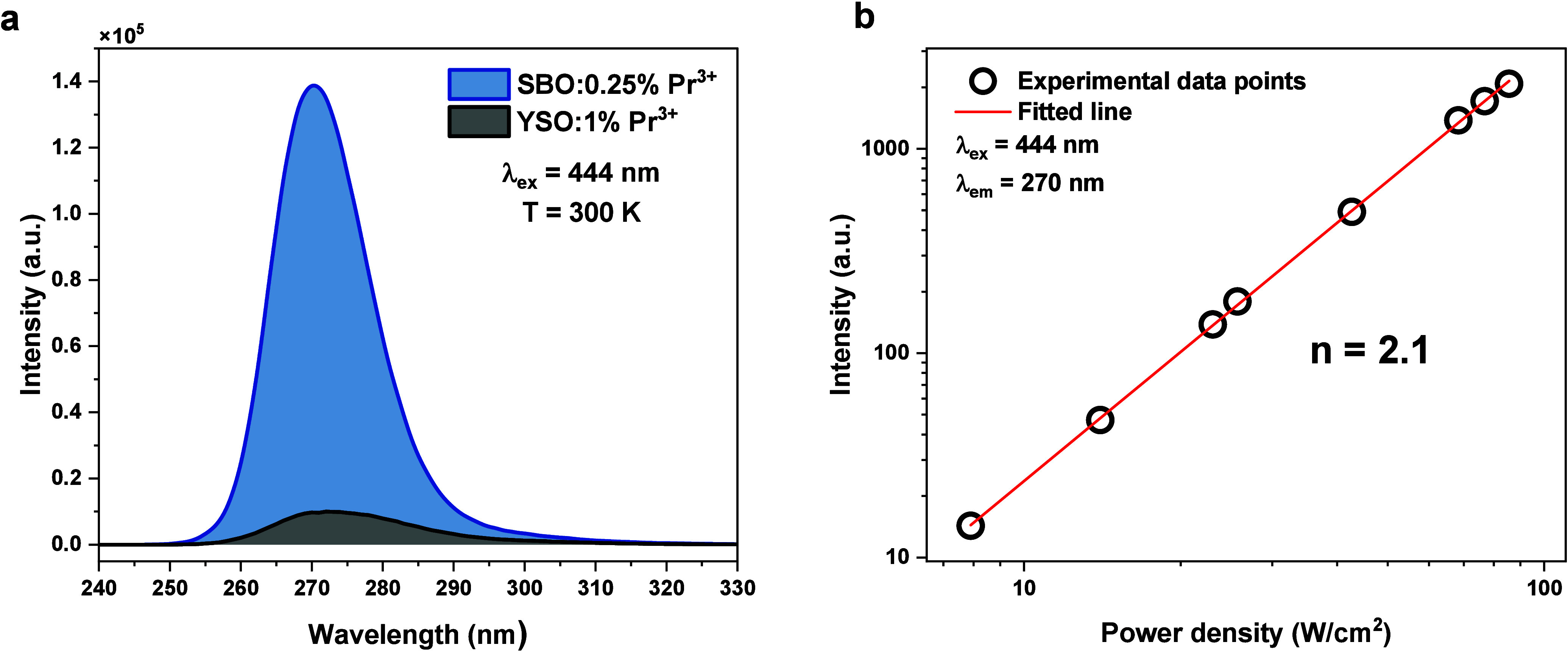
(a) Upconversion emission spectrum of SBO:0.25% Pr^3+^ (green line) and YSO:1% Pr^3+^ (red line) recorded under the same measurement conditions. (b) Power dependence of upconversion intensity at 270 nm measured under 444 nm continuous wave excitation.

The excitation with the blue light (441.6 nm) of course also generates emission in the visible region; however, the excitation from the ^3^P_0_ level is very weak, and the spectrum is dominated by transitions from the ^1^D_2_ level (see Figure 3a), which we confirmed measuring the emission spectra upon direct excitation (λ = 581.5 nm) into the ^1^D_2_ level (black line in Figure 3a). Interestingly, the lifetime of the ^3^P_0_ level is extremely short and decreases from 21 to 12 ns when the concentration increases from 0.1 to 2% (not presented here). On the other hand, the lifetime of the ^1^D_2_ level is much longer (34 μs) and is single-exponential. Moreover, we found that the lifetime of this level does not depend upon the Pr^3+^ concentration in the broad range of 0.1–7% (Figure 3b). That may indicate that, despite cross-relaxation (^1^D_2_, ^3^H_4_) → (^1^G_4_, ^3^F_2,3_), which usually very effectively quenches emission from this level, there must exist another mechanism that repopulates it. This issue will be addressed below in this letter.

**Figure 3:**
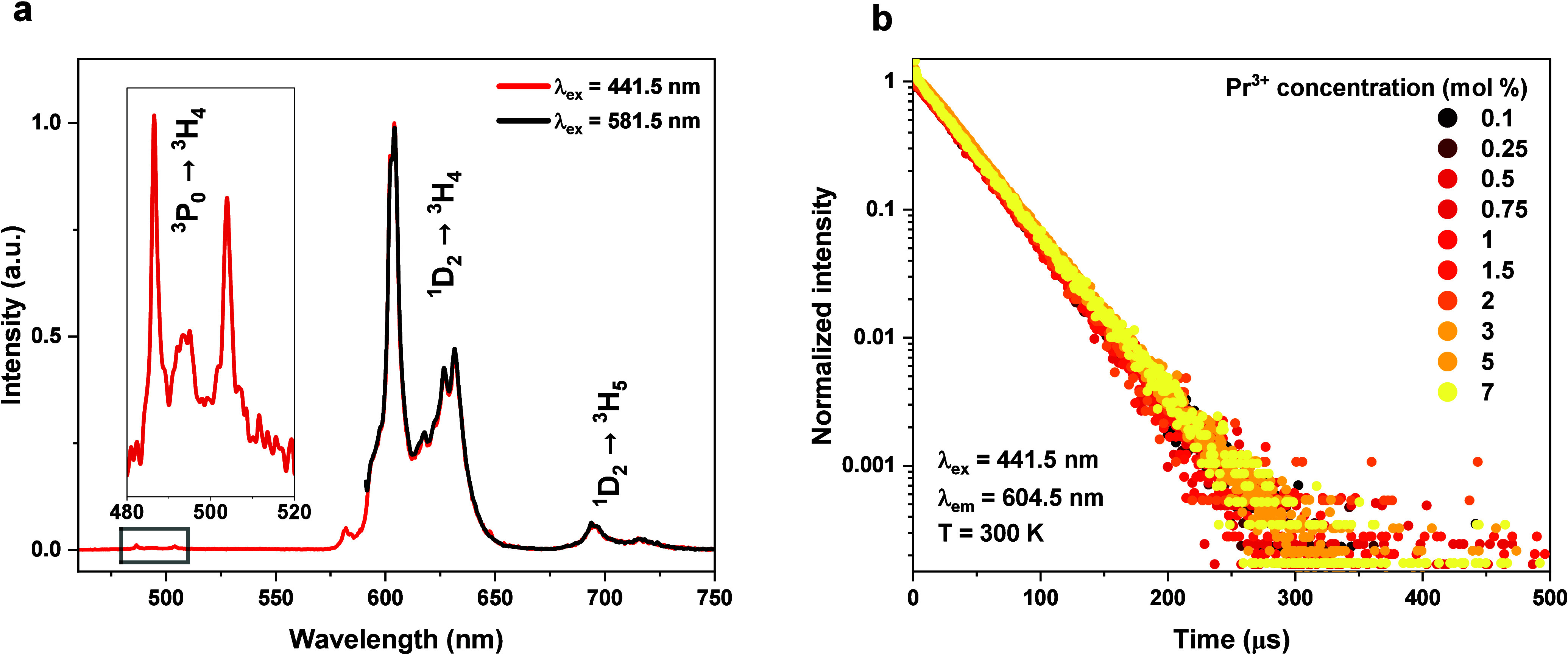
(a) RT Stokes emission spectra of SBO:0.25% Pr^3+^ excited at 441.5 nm (red line) and 581.5 nm (black line). The inset presents the part of the spectrum marked with the gray frame. (b) Decay kinetics of the ^1^D_2_ level (λ_ex_ = 441.5 nm and λ_em_ = 604.5 nm) of samples with different Pr^3+^ concentrations.

The mechanism responsible for the enhancement of the UC intensity proceeds in the following steps (see Figure 4). In the first, the excitation is absorbed by the ^3^P_2_ term and non-radiatively relaxes to the ^3^P_0_ level. The excited electrons do not stay long at the ^3^P_0_ emitting level due to the high phonon energies of the host. The latter is quickly emptied by (^3^P_0_, ^3^H_4_) → (^1^D_2_, ^3^H_6_) phonon-assisted cross-relaxation (CR) and ^3^P_0_ → ^1^D_2_ multiphonon relaxation (MPR), filling the ^1^D_2_ level. Note that it is difficult to directly excite the ^1^D_2_ level because the ^3^H_4_ → ^1^D_2_ transition is spin-forbidden. Moreover, the energy difference between the ^1^D_2_ and lower lying ^1^G_4_ levels is above 6500 cm^–1^, making quenching via MPR negligible. For both reasons, ^1^D_2_ is a metastable state and electrons stay there much longer than at the ^3^P_0_ level. This allows efficient pumping of the 5d electronic configuration via the parity-allowed ^1^D_2_ → 4f5d transition.

**Figure 4:**
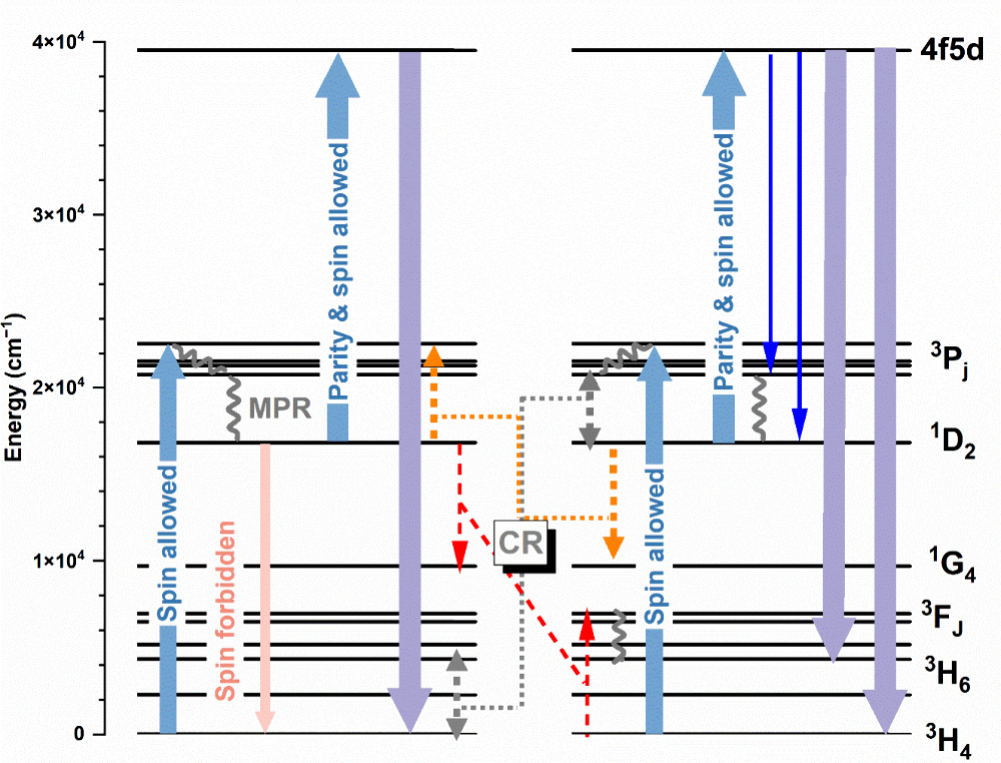
Energy level diagrams presenting the mechanism of vis-to-UVC upconversion occurring for the high phonon energy host doped with Pr^3+^ ions. For clarity, not all possible transitions are shown in the figure, and wavy lines represent non-radiative MPR transitions.

The invariance of the emission lifetime of the ^1^D_2_ level to Pr^3+^ concentration is unusual because an emission quenching is typically observed due to the strong CR process
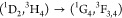
1the rate of which increases with an increasing concentration. Because the lifetime of the ^1^D_2_ level does not change with the dopant concentration, additional mechanisms must exist to populate this level. Such a process was proposed by Ganem et al., who studied the upconversion in Pr^3+^:YAG.^9^ They indicated that the UV fluorescent ion population is indirectly controlled by energy transfer processes involving ions in the ^1^D_2_ state. As a necessary condition for the effectiveness of the described mechanism, they emphasize an efficient energy transfer from the triplet; i.e., at least 50% of the ^3^P_0_ level population must decay to ^1^D_2_. In SBO, this condition is more than met, as ^3^P_0_ is almost completely emptied to give ^1^D_2_. The ^3^H_6_ level is radiatively populated, and this process is effective as it is observed from the emission (see also Figure 4). In conclusion, after upconversion and then the population of the ^3^H_6_ level, two CR processes are possible that repopulate the ^1^D_2_ level in a loop-like mechanism
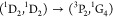
2which is phonon-assisted and
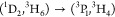
3The ^3^P_J_ term is drained directly to ^1^D_2_. For such a loop to work efficiently, both ^1^D_2_ and ^3^H_6_ must be populated for at least a dozen microseconds. As is known, the lifetime of the former meets this condition. However, due to the high phonon energies, the ^3^H_6_ level can be efficiently emptied by MPR. However, this level is characterized by a very long radiative lifetime, and even if MPR shortens its lifetime by 3 orders of magnitude still, the ^3^H_6_ population should be preserved with a lifetime of about a dozen microseconds. Of course, this hypothesis needs to be proven by additional experiments.

In our opinion, the efficiency of upconversion in SBO is also due to the conservation of spin in the ^1^D_2_ → 5d transition. According to Hund’s rule, the ground state of a given electronic configuration is the state with the highest spin number. In the case of Pr^3+^, the ground state of the 4f^2^ electronic configuration is the triplet ^3^H_4_ state. However, as Krośnicki et al.^10^ note, in the case of interconfigurational transitions, Hund’s rule must be treated with caution, as it may be broken due to spin–orbit interactions. According to their calculations for CaF_2_:Pr^3+^, the lowest level of the 4f^1^5d^1^(eg) configuration has 80% singlet character. The same observations for PrCl_3_ were obtained by Garcia and Faucher,^11^ who showed that the lowest ^S^L_J_ level of 4f^1^5d^1^ also has a singlet character. In another work, excited-state absorption spectra of three fluoride matrices KY_3_F_10_, LiYF_4_, and BaY_2_F_8_ doped with Pr^3+^ proved that the lowest level of the 4f^1^5d^1^ configuration has a more pronounced singlet character.^12^ As we will try to show in the next work using *ab initio* calculations, transitions from the ^1^D_2_ level to the upper 4f^1^5d^1^ configuration in SBO are allowed by not only the parity rule but also the spin selection rule; therefore, upconversion is so effective in this host.

On the other hand, fluoride matrices are characterized by a weaker splitting of the 5d configuration and a higher energy of their lowest component compared to other hosts.^13^ In many fluorides, the first level with a triplet spin character in the 5d configuration is located higher than the lowest level with a singlet spin character. The only metastable level in Pr^3+^-doped fluorides that can be useful in vis–UVC upconversion is ^3^P_0_ because ^1^D_2_ is almost empty. Therefore, the upconversion efficiency of one-color pumping is low in these hosts because the pump photons of around 450 nm possess too low of energy to reach the triplet state and are less absorbed by lower levels with a singlet character.

Additionally, the intensity of the upconversion luminescence also depends upon the differences in the equilibrium geometries of the 4f and 5d levels of potential energy in the configurational coordinate diagram, which is reflected by the Stokes shift between the 5d excitation and emission spectra. On the basis of the Stokes emission spectra in the UV and vis range (Figure 1), we can conclude that the Stokes shift is relatively small and the crossover relaxation between the lowest 5d and ^3^P_J_ parabolas is neglected in the case of this phosphor.

In borate Sr_3_(BO_3_)_2_ doped with Pr^3+^ ions, efficient MPR and phonon-assisted CR results in the creation of Pr^3+^ ions being in the metastable state ^1^D_2_, ready to absorb pump photons of 444 nm and efficiently transfer electrons to the 4f^1^5d^1^ configuration in parity and spin-allowed transitions. In the first step, the 444 nm excitation is absorbed in the ^3^H_4_ → ^3^P_2_ transition to fill the ^1^D_2_ level via CR and MPR. High phonon energies are not an obstacle to obtain efficient UVC upconversion emission, because the distance between the 4f5d levels and the ^3^P_J_ term is very large, far exceeding the five phonons capable of bridging this gap. Similarly, the ^1^D_2_ level is not efficiently quenched by MPR transitions, as the ^1^D_2_–^1^G_4_ distance is larger than 6500 cm^–1^ and the down CR from this level is compensated by a closed-loop-like mechanism, which has been proposed to explain its partial repopulation. More detailed studies on this system should be undertaken to prove it. We believe that high phonon energy materials, such as borates, silicates, and phosphates, will be the future of UVC UC phosphors.

## Supporting Information Available

Transparent Peer Review report available (PDF)
