# Supplementary material for: Discovering the Potential of High Phonon Energy Hosts in the Field of Visible-to-Ultraviolet C Upconversion
Source: J Phys Chem Lett. 2024 Sep 6;15(37):9356–60. doi: 10.1021/acs.jpclett.4c02053 (PMC11417987; doi:10.1021/acs.jpclett.4c02053)
Supplement: Supplementary file 1 — jz4c02053_si_001.pdf [file jz4c02053_si_001.pdf]

jz-2024-02053s.R1

Name: Peer Review Information for "Discovering the Potential of High-Phonon Energy Hosts in the Field of Vis-to-UVC Upconversion"

First Round of Reviewer Comments

Reviewer: 1

Comments to the Author
The authors present an extremely interesting new finding in the field of blue-to-UV upconversion with Pr^{3+} based on the host compound $Sr_3(BO_3)_2$. The findings are certainly novel and highly stimulating in the field of upconversion phosphors, which clearly make this work suited for publication in Journal of Physical Chemistry Letters.
Overall, this manuscript is well-written already and apart from little issues in English language (i.e. this should be checked again upon revision), only few aspects should be considered in more detail prior to final publication. Thus, only minor revision is necessary at this stage.

1) Instead of the abbreviation MNR, I would suggest using MPR (MultiPhonon Relaxation), which is more common in luminescence literature and thus, allows a clearer identification.

2) In Figure 2.(a), both a red and a green spectrum are depicted. For people having a disability to distinguish these two colors, it may be better to choose a different color such as blue instead of green.

3) How do the authors assess the issue that the (excited state absorption) transition from the 1D2 level to the lowest excited states of the 4f15d1 configuration with dominant spin triplet character by Hund's rules still works so well? Do they have an idea what could be the reason here?

4) A second issue is the comparably low decay time of the 1D2 level of only 34 µs, which is also given for the 3P0 level in several Pr^{3+} activated fluorides with a low phonon energy. What do the authors think is the reason for the yet so efficient upconversion process in this borate?

5) The authors propose an energy looping mechanism relying on the cross relaxation process [1D2, 3H6] → [3P1, 3H4]. What is the origin of the population of the 3H6 level of Pr^{3+}? How do they explain that? I do agree that the hypothesis is reasonable but this population is not so straightforward. If they argue by the previous [3P0, 3H4] → [1D2, 3H6] mechanism, this will dominantly depend on the decay time of the 3H6 level on the one hand, however, the 3P1 and 3P0 level have extremely low decay times in the ns range according to the authors. A few details and how they want to prove this hypothesis may be helpful for the general reader to justify this proposed mechanism.

6) In the conclusions section, the authors write that they "demonstrated a new closed-loop-like mechanism". I think it is more accurate to write that they propose a new closed-loop-like mechanism as their message is that they want to undertake additional more detailed studies on this system in future to prove this mechanism.

Author's Response to Peer Review Comments:

Wrocław 28 August 2024

Dear Reviewers,

We are very grateful for all the comments from the reviewers. It helped us improve the work and at the same time, the questions raised were very interesting. All issues were addressed and changes in the manuscript were highlighted.

Question

1) **Instead of the abbreviation MNR, I would suggest using MPR (MultiPhonon Relaxation), which is more common in luminescence literature and thus, allows a clearer identification.**

Answer:

Thank you for this comment, of course, the abbreviation MPR should be used. The text was corrected.

Question

2) **In Figure 2.(a), both a red and a green spectrum are depicted. For people having a disability to distinguish these two colors, it may be better to choose a different color such as blue instead of green.**

Answer:

Thank you for this comment, the colors in the Fig. 2a were changed.

Questions number 3 and 4:

Thanks for these comments (which are quoted below), we are responding to them in one reply.

3) **How do the authors assess the issue that the (excited state absorption) transition from the 1D2 level to the lowest excited states of the 4f15d1 configuration with dominant spin triplet character by Hund's rules still works so well? Do they have an idea what could be the reason here?**

4) **A second issue is the comparably low decay time of the 1D2 level of only 34 µs, which is also given for the 3P0 level in several Pr3+ activated fluorides with a low phonon energy. What do the authors think is the reason for the yet so efficient upconversion process in this borate?**

Answer:

These two questions are very interesting and concern topics that we believe have not been sufficiently studied. I think that such questions will arise in any attentive reader, so we decided to include in the manuscript the following paragraphs, which I hope will also answer the reviewer's questions.

In our opinion, the efficiency of upconversion in SBO is also due to the conservation of spin in the $^{1}D_2 \rightarrow 5d$ transition. According to Hund's rule, the ground state of a given electronic

configuration is the one with the highest spin number. In the case of Pr^{3+}, the ground state of the $4f^2$ electronic configuration is the triplet $^3H_4$ state. However, as Krośnicki et al. [11] note, in the case of interconfigurational transitions, Hund's rule must be treated with caution, as it may be broken due to spin-orbit interactions. According to their calculations for CaF_2:Pr^{3+}, the lowest level of the $4f^15d^1$(eg) configuration has 80% of singlet character. The same observations for $PrCl_3$ were obtained by Garcia and Faucher [11], who showed that the lowest ^SL_J level of $4f^15d^1$ also has singlet character. In another work, excited-state absorption spectra of three fluoride matrices KY_3F_{10}, $LiYF_4$, and BaY_2F_8 doped with Pr^{3+}, proved that the lowest level of the $4f^15d^1$ configuration has a more pronounced singlet character [12]. As we will try to show in the next work using ab initio calculations, transitions from the $^1D_2$ level to the upper $4f^15d^1$ configuration in SBO are allowed not only by the parity rule but also by the spin selection rule, therefore upconversion is so effective in this host.

On the other hand, fluoride matrices are characterized by a weaker splitting of the 5d configuration, and a higher energy of their lowest component compared to other hosts [13]. In many fluorides, the first level with triplet spin character in the 5d configuration is located higher than the lowest one with singlet spin character. The only metastable level in Pr^{3+} doped fluorides that can be useful in VIS - UVC upconversion is $^3P_0$ because the $^1D_2$ is almost empty. Therefore, the upconversion efficiency of one-color pumping is low in these hosts because the pump photons of around 450 nm possess too low energy to reach the triplet state and are less absorbed by lower levels with singlet character.

Additionally, the intensity of the upconversion luminescence depends also on the differences in the equilibrium geometries of 4f and 5d levels of potential energy in the configurational coordinate diagram, which is reflected by the Stokes shift between the 5d excitation and emission spectra. Based on the Stokes emission spectra in the UV and Vis range (Figure 1) we can conclude that the Stokes shift is relatively small, and the crossover relaxation between the lowest 5d and ^3P_J parabolas is neglected in the case of this phosphor.

References

[10] Krośnicki, M.; Kędziorski, A.; Seijo, L.; Barandiarán, Z. Ab initio Theoretical Study on the $4f^2$ and 4f5d Electronic Manifolds of Cubic Defects in CaF_2:Pr^{3+}. J. Phys. Chem. A **2014**, *118*, 358−368.

[11] Garcia, D. and Faucher M. A full calculation of multiconfiguration interaction effects up to 120000 cm^{-1} (15 eV) on the ground configuration state levels of $PrCl_3$. Zeeman effect interpretation, J. Chem. Phys. **1989**, *91*, 7461-7466.

[12]] Laroche, M.; Braud, A.; Girard, S.; Doualan, J.L.; Moncorge, R.; Thuau, M.; Merkle, L.D. Spectroscopic Investigations of the 4*f*5d Energy Levels of Pr^{3+} in Fluoride Crystals by Excited-State Absorption and Two-Step Excitation Measurements. *J. Opt. Soc. Am. B* **1999**, *16*, 2269–2277.

[13] Dorenbos, P. 5d-Level Energies of Ce^{3+} and the Crystalline Environment. I. Fluoride Compounds. *Phys. Rev. B* **2000**, *62*, 15640–15649.

Question:

5) The authors propose an energy looping mechanism relying on the cross relaxation process [1D2, 3H6] → [3P1, 3H4]. What is the origin of the population of the 3H6 level of Pr3+? How do they explain that? I do agree that the hypothesis is reasonable but this population is not so straightforward. If they argue by the previous [3P0, 3H4] → [1D2, 3H6] mechanism, this will dominantly depend on the decay time of the 3H6 level on the one hand, however, the 3P1 and 3P0 level have extremely low decay times in the ns range according to the authors. A few details and how they want to prove this hypothesis may be helpful for the general reader to justify this proposed mechanism.

Answer:

Thank you very much for this remark, indeed it needs to be explained better. We propose the following paragraphs to elucidate this issue Figure 4 has also been improved.

The invariance of the emission lifetime of the $^1D_2$ level to Pr^{3+} concentration is unusual because an emission quenching is typically observed due to the strong CR process:

$$(^1D_2, {}^3H_4) \rightarrow (^1G_4, {}^3F_{3,4}), \qquad (1)$$

the rate of which increases with increasing concentration. Since the lifetime of the $^1D_2$ level does not change with dopant concentration, additional mechanisms must exist to populate this level. Such a process was proposed by Ganem et al. who studied the upconversion in YAG: Pr^{3+} [9]. They indicated that the UV fluorescent ion population is indirectly controlled by energy transfer processes involving ions in the $^1D_2$ state. As a necessary condition for the effectiveness of the described mechanism, they emphasize an efficient energy transfer from the triplet, i.e. at least 50% of the $^3P_0$ level population must decay to $^1D_2$. In SBO this condition is more than met, as the $^3P_0$ is almost completely emptied to the $^1D_2$. The level $^3H_6$ is radiatively populated and this process is effective as it is observed from the emission. In conclusion, after upconversion and then the population of the $^3H_6$ level, two CR processes are possible that repopulate the $^1D_2$ level in a loop-like mechanism:

$$(^1D_2, {}^1D_2) \rightarrow (^3P_2, {}^1G_4) \qquad (2)$$

which is phonon-assisted, and

$$(^1D_2, {}^3H_6) \rightarrow (^3P_1, {}^3H_4) \qquad (3).$$

The ^3P_J term is drained directly to $^1D_2$. For such a loop to work efficiently both $^1D_2$ and $^3H_6$ must be populated for at least a dozen microseconds. As is known, the lifetime of the former meets this condition. But, due to the high phonon energies, the $^3H_6$ level can be efficiently emptied by MPR. However, this level is characterized by a very long radiative lifetime, and even if MPR shortens its lifetime by three orders of magnitude still, the $^3H_6$ population should be preserved with a lifetime of about a dozen microseconds. Of course, this hypothesis needs to be proven by additional experiments.

[9] J.Ganem et al. One-color sequential pumping of the 4f5d bands in Pr-doped yttrium aluminum garnet, J. Lumin. **1992**, 54, 79-87.

Question:

6) In the conclusions section, the authors write that they "demonstrated a new closed-loop-like mechanism". I think it is more accurate to write that they propose a new closed-loop-like mechanism as their message is that they want to undertake additional more detailed studies on this system in future to prove this mechanism.

Answer:

You are completely right, the conclusions were changed.

Similarly, the $^{1}D_{2}$ level is not efficiently quenched by MPR transitions, as the $^{1}D_{2}$–$^{1}G_{4}$ distance is larger than 6500 cm^{-1}, and down CR from this level is compensated by closed-loop-like mechanism which has been proposed to explain its partial repopulation. More detailed studies on this system should be undertaken to prove it.
